# Global change scenarios in coastal river deltas and their sustainable development implications

**DOI:** 10.1016/j.gloenvcha.2023.102736

**Published:** 2023-09

**Authors:** Murray W. Scown, Frances E. Dunn, Stefan C. Dekker, Detlef P. van Vuuren, Sitar Karabil, Edwin H. Sutanudjaja, Maria J. Santos, Philip S.J. Minderhoud, Ahjond S. Garmestani, Hans Middelkoop

**Affiliations:** a Lund University Centre for Sustainability Studies (LUCSUS), Lund, Sweden; b Copernicus Institute of Sustainable Development, Utrecht University, Utrecht, Netherlands; c Department of Physical Geography, Utrecht University, Utrecht, Netherlands; d Department of Earth Sciences, Utrecht University, Utrecht, Netherlands; e PBL Netherlands Environmental Assessment Agency, The Hague, Netherlands; f Institute for Marine and Atmospheric Research Utrecht (IMAU), Utrecht University, Utrecht, Netherlands; g Department of Geography, University of Zurich, Zurich, Switzerland; h Wageningen University and Research, Wageningen, Netherlands; i Department of Civil, Environmental and Architectural Engineering, University of Padova, Padova, Italy; j Department of Subsurface and Groundwater Systems, Deltares Research Institute, Utrecht, Netherlands; k Utrecht Centre for Water, Oceans and Sustainability Law, Utrecht University School of Law, Utrecht, Netherlands; l Office of Research and Development, US Environmental Protection Agency, Gulf Breeze, FL, United States

**Keywords:** Sea-level rise, Climate risk, Sustainable development, Urbanisation, SSP, RCP

## Abstract

Deltas play a critical role in the ambition to achieve global sustainable development given their relatively large shares in population and productive croplands, as well as their precarious low-lying position between upstream river basin development and rising seas. The large pressures on these systems risk undermining the persistence of delta societies, economies, and ecosystems. We analyse possible future development in 49 deltas around the globe under the Shared Socio-economic and Representative Concentration Pathways until 2100. Population density, urban fraction, and total and irrigated cropland fraction are three to twelve times greater in these deltas, on average, than in the rest of the world. Maximum river water discharges are projected to increase by 11–33 % and river sediment discharges are projected to decrease 26–37 % on average, depending on the scenario. Regional sea-level rise reaches almost 1.0 m by 2100 for certain deltas in the worst-case scenario, increasing to almost 2.0 m of relative rise considering land subsidence. Extreme sea levels could be much higher still—reaching over 4.0 m by 2100 for six of the 49 deltas analysed. Socio-economic conditions to support adaptation are the weakest among deltas with the greatest pressures, compounding the challenge of sustainable development. Asian and African deltas stand out as having heightened socio-economic challenges—huge population and land use pressures in most Asian deltas and the Nile delta; low capacity for adaptation in most African deltas and the Irrawaddy delta. Although, deltas in other parts of the world are not immune from these and other pressures, either. Because of unique pressures and processes operating in deltas, as in other “hotspots” such as small islands, mountains, and semi-arid areas, we recommend greater consideration and conceptualisation of environmental processes in global sustainable development agendas and in the Integrated Assessment Models used to guide global policy.

## 1. Introduction

Coastal river deltas are places of concern for global sustainable development. Within deltas, over 5 % of the world’s population lives on less than 0.5 % of the Earth’s land (Dunn et al., 2019). Population densities rise well over 10,000 people per square kilometre in urban parts of some African and Asian deltas (Ericson et al., 2006), and other deltas are undergoing rapid urbanisation, amplifying the need to guide prudent urban development in these places. Deltas are also important places for food security and global trade because of their highly productive agricultural lands and the access to resources via waterways (Evans, 2012; Syvitski, 2008), but the persistence of such flows of environmental goods provided by deltas is at risk (Reader et al., 2022). At the same time, the low elevation of deltas means that they are highly exposed to risks from relative sea-level rise, flooding, and salinisation. As a result of these risks and further urbanisation, ecosystems in deltas are increasingly degraded or lost (Giosan et al., 2014; Syvitski, 2008). The importance of deltas for development combined with the high environmental risks form an important argument for the need for achieving the United Nations’ Sustainable Development Goals (SDGs) in these places and globally (Scown, 2020; Szabo et al., 2016).

Environmental change relevant for long-term delta sustainability occurs at multiple scales (Day et al., 2016; Santos and Dekker, 2020). Globally, sea-level rise places pressure on these environments (Alley et al., 2005). At the drainage basin scale, dams for hydropower development and water consumption lead to a disconnection of the delta from its upstream area, depriving it of river sediment (Dunn et al., 2019; Schmitt et al., 2018). Within deltas, human activities cause local effects such as subsidence due to extraction of groundwater and hydrocarbons, land drainage, and construction (Syvitski et al., 2009). Exploitation of deltas to yield social and economic benefits has led to many deltas becoming “locked-in” to states of high risk (Santos and Dekker, 2020) and costly, rigid, and energy-intensive adaptation strategies (e.g., the Dutch Delta Works). Socio-economic systems in many deltas have become disconnected from the ecosystems upon which they fundamentally rely (Reader et al., 2022). These pressures and states of deltas have inevitable consequences for their sustainability and the SDGs.

While the importance of deltas for sustainable development is well-established (Nicholls et al., 2019; Szabo et al., 2016) and scholars have proposed measures of delta risk (Tessler et al., 2015) and sustainability (Day et al., 2016), future developments in deltas around the globe under different scenarios, and across scales, remain highly uncertain and unexplored. Globally, previous assessments of future risk to deltas have been done from a single perspective—e.g., sediment starvation (Dunn et al., 2019), land loss (Nienhuis et al., 2023; Nienhuis and van de Wal, 2021), or population exposure to flooding (Nicholls et al., 2021), which neglects other dimensions of delta social-ecological systems that affect risk—or by creating rudimentary future scenarios in which only energy prices change (Tessler et al., 2015), which does not capture alternative trajectories of population growth, land-use change, sea-level rise, or socio-economic development over the coming century. Locally in individual deltas, Integrated Assessment Models have been used to analyse trade-offs among SDGs under different future scenarios (e.g., Hutton et al., 2018), but their widespread application to many deltas globally has not yet been achieved.

**Fig. 1.**
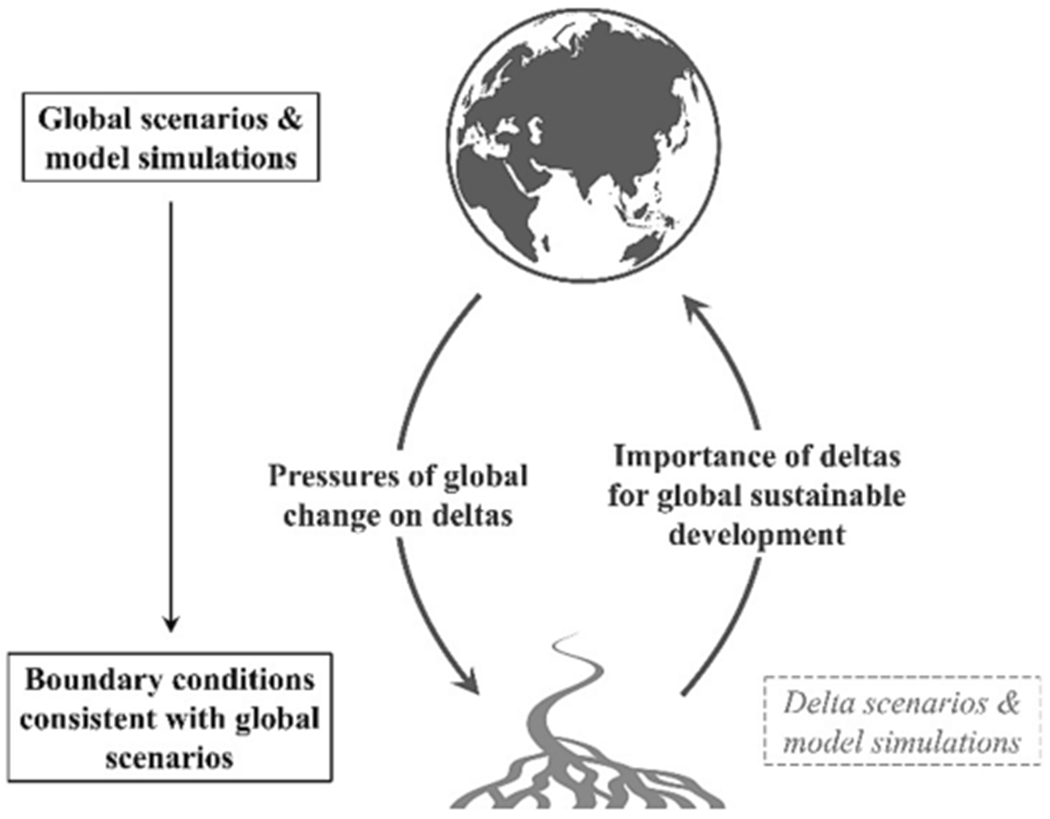
Cross-scale interactions between global and delta development. We determine boundary conditions on delta development that are consistent with global scenarios and model simulations. These boundary conditions reveal deltas most at risk under different scenarios, the importance of different global change drivers of risk, and can be used to inform local scenarios and model simulations in individual deltas.

The issue of scale pervades both delta risk and sustainable development (Scown et al., 2023; Wilbanks, 2007) and here we take a first step to bring the global to the local in deltas (Fig. 1). Global environmental change and socio-economic development will place local pressure on deltas, regardless of which possible scenario becomes a reality. Developments in many environmental and social dimensions have been simulated and estimated globally for a range of scenarios, and information regarding the pressures these changes will place on deltas is required to set boundary conditions for delta-scale scenarios and model simulations (e.g., Hutton et al., 2018). Such boundary conditions set by global changes should not be determined individually for deltas, but should be consistent with global scenarios across all deltas. At the same time, challenges faced in deltas—if not effectively navigated—could scale up to affect global development and the achievement of the SDGs. Therefore, assessment of possible development and identification of risks in deltas globally under a range of scenarios is of utmost importance.

To explore the pressures placed on a set of 49 deltas in the context of global sustainable development, we analyse how these deltas might change under different future scenarios of global change. We reveal the disproportionate pressures on these deltas, which we argue will affect achieving the SDGs across multiple scales. We show, using the global Shared Socio-economic Pathways (SSPs) (O’Neill et al., 2017; Riahi et al., 2017) and Representative Concentration Pathways (RCPs) (van Vuuren et al., 2011), that future pressures on these deltas are highly divergent under different scenarios, and that Asian and some African deltas face heightened challenges for sustainable development. We discuss issues of institutional fit, scale, and feedbacks, which require careful consideration in pursuit of the SDGs. We recommend a greater conceptualisation of “the environment” in future global sustainable development agendas by focusing on “hotspots” that are based not only on societal needs but also on environmental context. This will require a longer-term horizon for development agendas in line with global climate and environmental change, as well as a consideration of cross-scale interactions in coupled social-environmental systems (Scown et al., 2023).

## 2. Material and methods

**Fig. 2.**
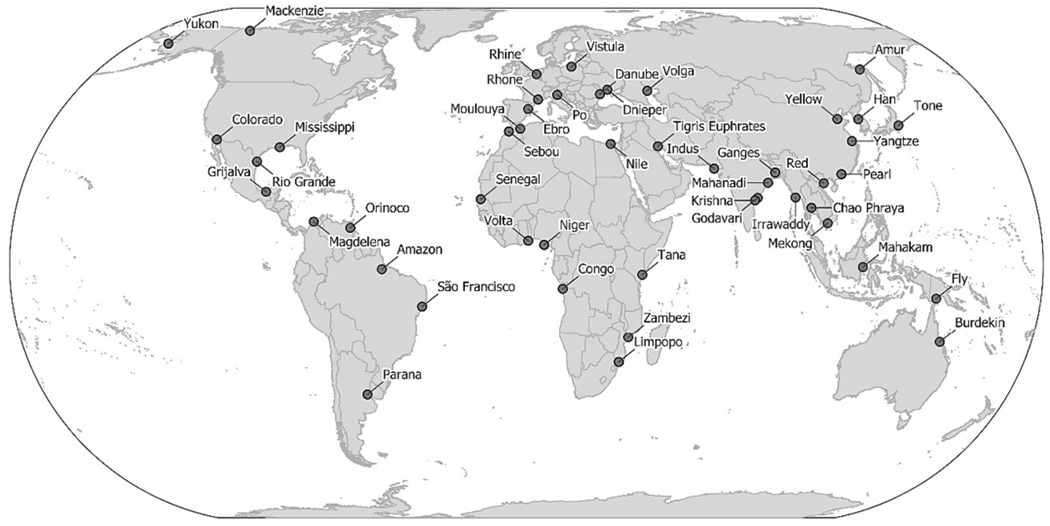
Location of 49 deltas analysed in this study.

For this study we explored future scenarios for 49 deltas, including the most populated and largest coastal deltas in the world, as well as a range of smaller and less populated deltas representing different climates, biomes, and socio-economic development states (Fig. 2). The set of deltas corresponds to only a fraction of the total number of deltas globally (cf. 235 large deltas of Reader et al., 2022; and the thousands of deltas of Nienhuis et al., 2020), but were chosen to represent a wide range of geographies and align with previous work on delta risk (Dunn et al., 2019; Tessler et al., 2015) and the availability of limited data on land subsidence (Nicholls et al., 2021). For our analysis we chose a set of 13 variables that represent various socio-economic and geophysical pressures on deltas and are indicators of the different components of delta risk as defined by Tessler et al. (2015) (Table 1).

The indicators were analysed under a range of future global scenarios reflecting the narratives of the SSPs (O’Neill et al., 2017, 2014) and the RCPs (van Vuuren et al., 2011). SSP1 is a sustainable development scenario with low challenges for climate change mitigation and adaptation because of increased environmental awareness, reduced inequality, and less resource-intensive lifestyles (O’Neill et al., 2017). By contrast, SSP3 describes a future with high climate mitigation and adaptation challenges due to rapid population growth, slow technological change, high inequalities, and weak institutions (O’Neill et al., 2017). In between these two extremes, SSP2 represents a “middle of the road” scenario in which future development is typical of historic trends, resulting in intermediate climate change mitigation and adaptation challenges (O’Neill et al., 2017). SSP4 is a scenario of inequality creating high climate adaptation challenges but low mitigation challenges, while development in SSP5 is driven by fossil fuels creating high mitigation challenges but lower adaptation challenges due to wealth creation. Alongside the SSPs, we explored associated scenarios correspond with low, medium, and high scenarios of sea-level rise during this century: RCP2.6 (low, maximum mitigation), RCP4.5 and RCP6.0 (midrange, stabilisation of GHG concentrations), and RCP8.5 (extreme, growing emissions). We note that RCP8.5 is representative of very high levels of temperature change and is not representative for best-guess representations of reference scenarios (Hausfather and Peters, 2020). For consistency, in our analysis we linked SSP and RCP scenarios as in the Sixth Assessment Report of the Intergovernmental Panel on Climate Change (IPCC).

The extents of the 49 deltas were based on the geospatial delta shapefiles of Tessler et al. (2015) with minor modifications conducted by Dunn et al. (2019). The shapefiles were converted to 5 arcminute and 1/8th degree rasters to align with model output grids, using the “maximum combined area” method in ArcGIS 10.6. Spatial averages of each variable were calculated using the raster package in R. For the final analyses of individual delta population and land use indicators, the following nine small deltas occupying fewer than 10 grid cells at 1/8th degree resolution were excluded: Burdekin, Dnieper, Ebro, Moulouya, Po, Sebou, São Francisco, Tana, and Tone (Fig. 2). Still, these small deltas were retained for the aggregated global comparison between “deltas” and “non-deltas”, as well as for sea level, economic, and governance indicators. For these 49 deltas we determined the indicators listed in Table 1 as follows:

To analyse how population density and land use may create pressure on deltas under each scenario, we used gridded global model outputs projected until 2100. Global gridded population projections at 1/8th degree resolution (approximately 14 × 14 km^2^ at the equator) were taken from Jones and O’Neill (2016). The urban land fraction was calculated from Gao and O’Neill (2020), also at 1/8th degree resolution. Irrigated and total cropland fractions at 5 arcminute resolutions (approximately 9 × 9 km^2^ at the equator) were taken from the Integrated Assessment Model IMAGE 3.0 (Doelman et al., 2018; Stehfest et al., 2014). We assume that cropland requirements in deltas must be met, so any increase in urban area must come from other land.

We summarised the gridded model outputs for four pressure variables—population density, urban land fraction, total and irrigated cropland fractions—in “delta” (within the 49 deltas combined extent) and “non-delta” (outside the 49 delta extents and excluding land covered by permanent ice or water) areas worldwide, as well as within each of the individual deltas (Fig. 2). While analysis of our entire set of deltas reveals disproportionate pressures on these systems and their importance for global sustainable development, focusing on individual deltas reveals those where certain issues are most pertinent and urgent.

We present population change as a density and land use change as a fraction of the total area within each delta. These indicators could be used as boundary conditions for future delta-scale simulation modelling, but in themselves also reveal different pressures on deltas under different scenarios. We do not present gridded maps of population nor categorical land use over time because of the complexities of spatial dynamics in individual deltas; for example, channel migration, shoreline change, and surface hydrology. However, these density/fractional data could be calibrated with current delta-specific realities (e.g., from remote sensing or land use maps) and dynamically simulated to create delta-specific maps in the future. This is beyond our scope, but we provide the data for other users.

Scenarios for future fluvial sediment input to the deltas were taken from projections by WBMsed (Dunn et al., 2019), a spatially-distributed global hydrogeomorphic model run at 6 arcmin for the future scenarios, produced by combining three RCPs with SSPs 1–3 (i.e., RCP2.6-SSP1; RCP4.5-SSP2; and RCP6.0-SSP3). We acknowledge these combinations do not cover the full suite of RCPs and SSPs but we find them sufficient to demonstrate a broad range of possible future development pathways. Annual sediment flux data was taken from the point (grid cell) at which the river enters the delta area, which was calculated based on the geospatial delta extent shapefiles. For deltas with more than one major contributing river, the sediment fluxes from each river were summed. The data are presented as 30-year averages over the preceding decades.

For regional sea-level rise (SLR) we used the model ensemble from the IPCC’s Special Report on the Ocean and Cryosphere in a Changing Climate (SROCC) (Pörtner et al., 2019). We first aggregated the 1/8th degree delta mask to a resolution of 1 degree, then created a 3-degree buffer (7 × 7 cell moving window) around each delta to ensure overlap with cells in the SROCC SLR grid. We then calculated the average of the model ensemble median SLR in cells within this buffer for each delta. We experimented with buffers of 1, 2, 3, and 5 degrees around deltas and found negligible differences. The buffers were used to ensure we only captured cells nearby the coast of deltas in their SLR calculations.

Sea-level change estimates provided by the IPCC SROCC model ensemble are driven by the effects of global climate change (e.g., Greenland ice mass loss, thermal expansion of oceans) simulated by coupled climate models. Vertical land movements driven by tectonics and glacial isostatic adjustments following the last glacial maxim are not included. Global estimates of tectonic uplift suggest that “*most coastal segments have risen relative to sea-level with a mean uplift rate higher than 0.2 mm/y*” (Pedoja et al., 2011, p. 1), and uplift rates as high as 4 mm/y are estimated for the west coast of North America and parts of Southeast Asia (see Fig. 3 in Pedoja et al., 2011). Similarly, estimates of glacial isostatic adjustment in the Earth’s crush indicate a vertical rise of more than 10 mm/y in extreme areas such as around Hudson Bay, Canada (Peltier et al., 2018). Conversely, other parts of the crust are subsiding faster than 0.5 mm/y, particularly around the Atlantic coastlines of North America, Europe, and West Africa, the Caribbean, and the west coast of North America (Peltier et al., 2018). Our analyses do not take these vertical land movements into account.

We do, however, account for land subsidence within deltas. By nature, deltas are prone to subside, as they consist of young, unconsolidated sediments. Land subsidence is the cumulative effect of a range of natural processes, such as isostacy, tectonics and natural sediment compaction, and anthropogenic-driven processes, of which excessive drainage causing peat oxidation and aquifer-system compaction following groundwater over-pumping are the most prominent (Shirzaei et al., 2020). Together these drivers, especially the human-driven ones, can create highly variable subsidence patterns within a single delta (Candela and Koster, 2022), both spatially and in time with accelerations and decelerations (e.g., Minderhoud et al., 2018). Moreover, Nicholls et al. (2021) concluded that relative SLR currently experienced by coastal populations around the world is predominantly caused subsidence rather than global SLR (Nicholls et al. 2021), underscoring the importance of including subsidence in our deltaic SLR assessment.

For most deltas in the world no or limited data is available on subsidence, let alone detailed information of its spatial or temporal variability. There are a few noteworthy exceptions: the Mississippi delta, with an extensive monitoring network providing detailed spatial insights in shallow subsidence dynamics (Jankowski et al., 2017), the Rhine-Meuse delta with ample observations and high-resolution 3D geological models (e.g., Koster et al., 2018) and the Mekong delta, where spatial and temporal subsidence patterns were linked to land use and land-use change (Minderhoud et al., 2018) and projections of future subsidence were computed for different groundwater extraction scenarios (Minderhoud et al., 2020). In our assessment land subsidence rates were adopted from Nicholls et al. (2021) for 47 of the 49 deltas, who used estimates by, among others, Ericson et al. (2006) and, when not available, assumed a standard “expert judgement” value of 1.0 mm/y. This “expert judgement” was provided from the original source (Nicholls et al., 2021) and we do not interpret it further here. For the remaining Volga delta (Russia/Razakhstan) we also assumed 1.0 mm/y—for consistency with Nicholls et al. (2021)—as no data was available, and for the Po delta (Italy) we used the average value of 6.0 mm/y reported by Cenni et al. (2021). In our assessment we keep the subsidence rate constant in space and time for each delta and accumulate this from 2000 on top of regional SLR until 2100. We acknowledge that using a single constant value of subsidence is a strong simplification of actual subsidence dynamics in deltas. Ideally, future subsidence in a delta is included dynamically, depending on economic development and management measures as demonstrated by Minderhoud et al. (2020) for the Mekong delta, by projecting spatial and temporal variable subsidence in a similar way as SLR is projected using different RCP’s scenarios; however, this is not yet possible for most of the deltas we include.

Extreme sea levels were taken from Kirezci et al. (2020). Here, extreme sea level refers to the combined effects of tide, storm surge, wave setup, and regional SLR, accounting for the statistical probability of extreme events and the relative timing of high tides and storms (please see Kirezci et al., 2020, for full details). This data contains 1 in 100 year extreme sea levels in m for the “current” situation, and the years 2050 and 2100 for RCPs 4.5 and 8.5. For each delta, we take the average of all simulated sites within 1 degree of the delta (buffered on delta polygons in QGIS). We assume “current” to be 2020.

The maximum daily river water discharge projections were produced by the global hydrological model PCR-GLOBWB (Sutanudjaja et al., 2018), which runs at a 5 arc-minute and daily resolution. We used the PCR-GLOBWB output produced in the Aqueduct Flood Analyzer Project (Ward et al., 2020), for four RCPs (2.6, 4.5, 6.0, and 8.5) and for the climate models GFDL-ESM2M, HadGEM2-ES, IPSL-CM5A-LR, MIROC-ESM-CHEM, and NorESM1-M from the Inter-Sectoral Impact Model Intercomparison Project (ISIMIP; Hempel et al., 2013; McSweeney and Jones, 2016). The maximum daily water discharge per 30 years was taken from each model run to indicate projected changes in flood risk, and the median of the climate models is presented here. The water data was taken from the point (grid cell) at which the river enters the delta, calculated from the geospatial delta extent shapefiles as for the sediment analysis. For deltas with more than one major contributing river, the discharge was taken from the largest river. It was not possible to account for whether maximum daily discharges temporally coincide with extreme storm- and tide-driven sea levels.

To explore socio-economic conditions in the country or countries where each delta is located under different SSPs, we analysed future GDP, GDP per capita, government effectiveness (from the World Bank’s Worldwide Governance Indicators; World Bank, n.d.), and adaptation readiness (from the Notre Dame Global Adaptation Initiative (ND-GAIN); Chen et al., 2015). Countries are normally responsible for governing/investing in their deltas and the indicators used reflect the capacity of national government to implement effective adaptation. We assume that country-level GDP is positive for investment capacity (as per Tessler et al. (2015)); however, GDP or capital accumulation in deltas may also play a negative role by increasing economic exposure to hazards. Aggregate GDP is calculated for each country from the OECD GDP projections (Dellink et al., 2017) and GDP per capita is calculated from this using the IIASA population projections (Samir and Lutz, 2017), all of which are available in the SSP Public Database Version 2.0. The World Bank’s government effectiveness indicator captures “perceptions of the quality of public services, the quality of the civil service and the degree of its independence from political pressures, the quality of policy formulation and implementation, and the credibility of the government’s commitment to such policies” (Kaufmann et al., 2010, p. 4), which we assume affects the capacity of a government to formulate and implement risk management and adaptation strategies within a delta. The ND-GAIN adaptation readiness indicator is a composite of two dimensions affecting the capacity to implement adaptation strategies: the economic readiness and the government readiness (Chen et al., 2015). Economic readiness relates to a country’s ease of doing business, which affects whether the country can attract adaptation investments (Chen et al., 2015). Governance readiness is comprised of four factors. Three of these are included because of their effect on foreign investments, including adaptation investments (Chen et al., 2015): 1) political stability and non-violence, 2) control of corruption, and 3) rule of law. The fourth, regulatory quality, affects the development and deployment of adaptation policies and actions (Chen et al., 2015). Future government effectiveness and adaptation readiness under the SSPs are taken from Andrijevic et al. (2020). For deltas that span multiple countries, we take the spatially-weighted average of those countries based on the fraction of the delta they occupy. We acknowledge that “country” does not equal “delta”—our approach does not account for within-country heterogeneity and inequality. Sub-national historical estimates of GDP and human development are available (Kummu et al., 2018), but still remain to be developed for deltas under future scenarios.

All geospatial processing details, code, and final result outputs are provided in the Supplementary material.

## 3. Results

### 3.1. The 49 deltas compared to the rest of the ice-free land surface

**Fig. 3.**
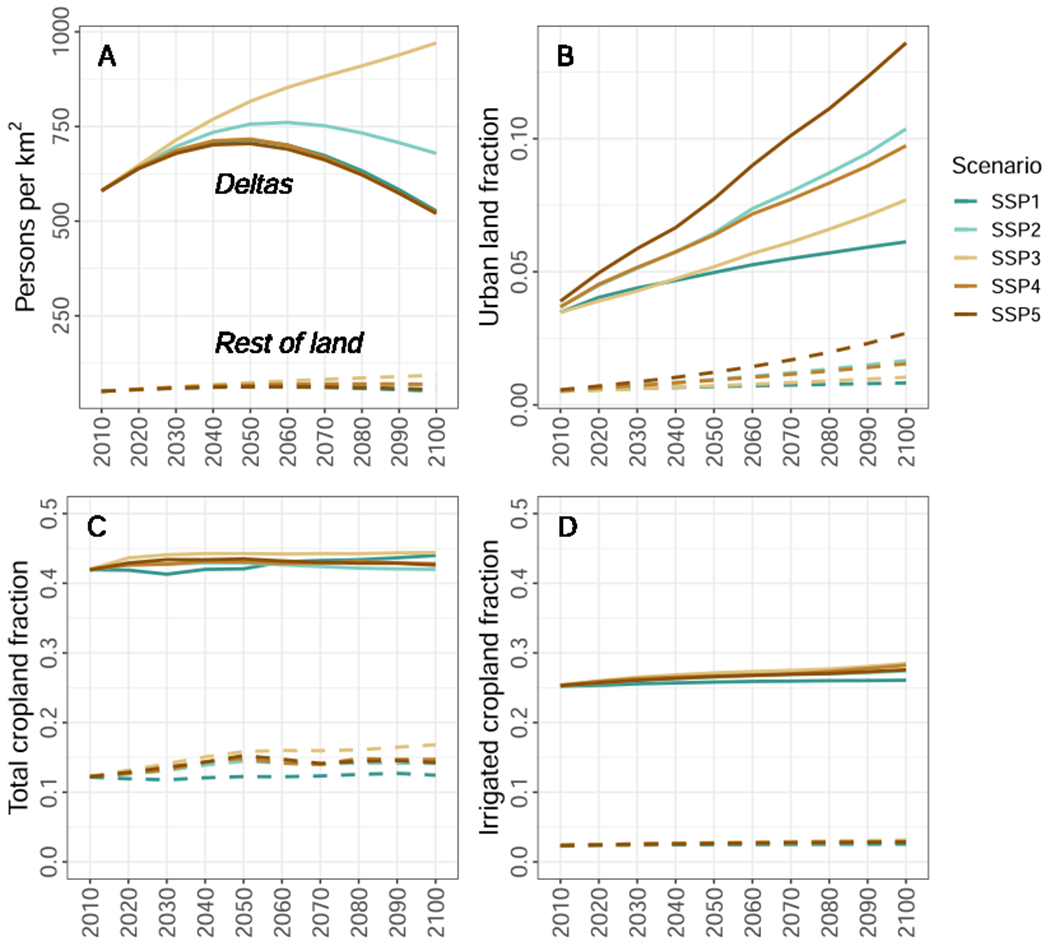
Disproportionate pressures of population density (A), urbanisation (B), total cropland (C), and irrigated cropland (D) in the 49 deltas, on average, compared to the rest of the world. Modelled scenario outputs downscaled to the grid level are shown as solid lines within the extents of the 49 deltas analysed and as dashed lines for the rest of the ice-free land surface (Rest of land) (see Materials and Methods for full details). Trends in these four indicators relative to 2010 are provided in the Supplementary material.

The population and land-use pressures on these 49 deltas are disproportionally large when compared to other ice-free land areas, which is likely to continue under a range of possible future scenarios (Fig. 3). Average population density across the 49 deltas is already almost 12 times higher (around 639–648 persons per km^2^ in 2020) than other ice-free land areas (around 55–56), barely easing to around 10 times higher by 2100 under all scenarios (Fig. 3A). Associated with high population densities, the fraction of urbanised delta land is currently, on average, seven times higher (around 4–5 % of the delta area in 2020, depending upon estimate) than other land areas (below 1 %), and global trends of urbanisation under all future scenarios are amplified in the deltas analysed (Fig. 3B). Similarly, our set of deltas have (on average and relative to their size) over three times as much cropland and almost 11 times as much irrigated cropland when compared to the rest of the world (Fig. 2C and D). The differences between scenarios for population density and urbanisation in deltas are striking, particularly when looking beyond the 2030 horizon of the SDGs: under the SSP3 scenario population growth is largest, with large populations remaining in rural areas; under SSP5 population declines and concentrates in urban areas leaving a very low rural population; whereas under SSP1 both population decreases with lower growth of urbanisation (Fig. 2A and B). Relative change in the areas of cropland and irrigated cropland in deltas is small under all scenarios, in contrast to the relative increase of more than 30 % in other ice-free land areas under SSP3 (Fig. S1G and H), suggesting that these land uses are already at capacity in these 49 deltas (on average) and are required in the future, but there is limited space for cropland expansion. Thus, allocation of urban land (which does increase with a similar trend between deltas and other ice-free land areas; Fig. S1F) must be at the expense of other land (e.g., forest), which may place further environmental pressure on these systems.

### 3.2. Possible futures of individual deltas

The figures and trends in Fig. 2 are averages over all 49 deltas analysed; in this section we show that the global models indicate considerable differences between the deltas, both for the current situation and possible future developments under each scenario.

#### 3.2.1. Population and land use in deltas

**Fig. 4.**
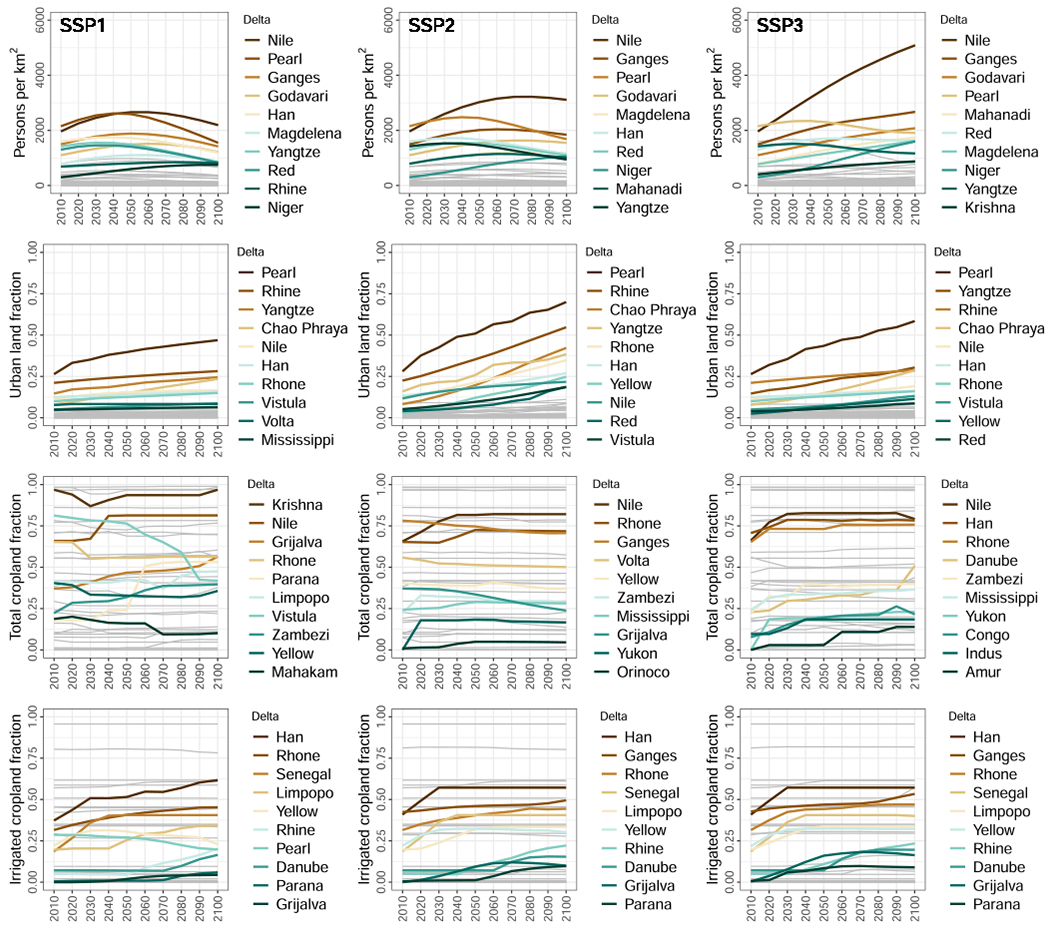
Population and land-use pressures on individual deltas under three future development scenarios. By row (top to bottom): population density, urban land fraction, total cropland fraction, and irrigated cropland fraction. By column (left to right): SSP1, SSP2, and SSP3. For population density and urban fraction, the top ten deltas in 2100 are highlighted for each plot. For cropland, the ten deltas with greatest change are highlighted, coloured by amount in 2100 (the top ten deltas are highlighted in the Supplementary material). All other deltas are shown as grey lines. Each indicator is calculated for each delta from global gridded model outputs at resolutions of 1/8th degree (population and urban) or 5 arcminutes (cropland). See Methods for full details and Supplementary material for all SSPs.

In terms of population density, Asian and some African deltas stand out as the highest under all scenarios analysed, along with the Magdalena in Colombia and the Rhine in The Netherlands (Fig. 4). The highest delta-average population densities are consistently seen in the Nile delta (Egypt), rising over 5,000 persons per square kilometre by 2100 under SSP3. Still, local population densities within a single delta can be much higher than the delta-average densities shown in Fig. 4. The Nile shows the most extreme increase in population density, in terms of absolute numbers, under SSP3 (Fig. 4); while under SSP1 and SSP2, population density in the Niger delta (Nigeria) more than doubles by 2100, despite being lower than other top deltas in absolute terms (Fig. 4). While delta populations reflect regional population trends in the simulated scenarios (Jones and O’Neill, 2016), deltas themselves face a unique suite of environmental pressures that can place their populations at much higher exposure to hazards (e.g., floods) than societies in other areas within the same region, likely having consequences for sustainable development.

The Pearl delta (China) is currently the most urbanised and remains so under all future scenarios, followed by the Rhine, Yangtze (China), and Chao Phraya (Thailand) (Fig. 4). Future urbanisation of many deltas is particularly pronounced under the “middle of the road” scenario (SSP2; Fig. 4), with the Pearl rising to almost 70 % urban by 2100 under this scenario, and even more so under SSP5 where the Pearl and Rhine both exceed 75 % by 2100 (Fig. S2). The most extreme expansion of urban area is seen in the Red (Hong) delta (Vietnam) under SSP5, with a seven-fold increase from around 4 % in 2010 to 28 % in 2100 (Fig. S2). The Chao Phraya delta also shows a more than six-fold increase in urban area by 2100 under SSP5. Increasingly high population and urbanisation in deltas places increasing numbers of people and infrastructure at risk from hazards, particularly flooding, which in the longer term—with progressive pressures from climate and sea-level rise—may dramatically hamper fulfilling the SDGs that relate to reducing the human and economic impacts of disasters after the year 2030. Additionally, urban development disrupts natural delta processes, such as flood retention in wetlands, and the weight of buildings and infrastructure can exacerbate land subsidence, further amplifying risks in these places.

In terms of agricultural land use, there is considerable demand for the productive land in deltas now and in the future under all scenarios (Figs. 3 and S3). Several deltas are already saturated (or close to) with cropland upon which large populations are dependent for food, including the Krishna, Godavari, and Mahanadi/Brahmani deltas in India and the Red delta in Vietnam (Fig. S3). The Red and Yangtze deltas are almost entirely used for irrigated agriculture (Fig. S3), which poses its own challenges for sustainable development in these places. In terms of change in cropland area, most deltas remain relatively stable over time under all scenarios (grey lines in Fig. 4). Those deltas currently mostly occupied by cropland remain so, indicating that the risks associated with delta agriculture also persist. The greatest fractional change in total cropland is, in fact, a decline from 81 % in 2010 to 42 % in 2100 in the Vistula delta (Poland), followed by an increase from 18 % to 53 % in the Paraná delta (Argentina and Uruguay), both under SSP1 (Fig. 4). The greatest increases in irrigated cropland fraction are seen for the Han delta (North and South Korea; 37 % to 62 %) under SSP1, the Senegal delta (Senegal; 19 % to 40–41 %) under all scenarios, and the Rhine (Netherlands; 5 % to 24 %) under SSP5.

#### 3.2.2. Geophysical pressures on deltas

**Fig. 5.**
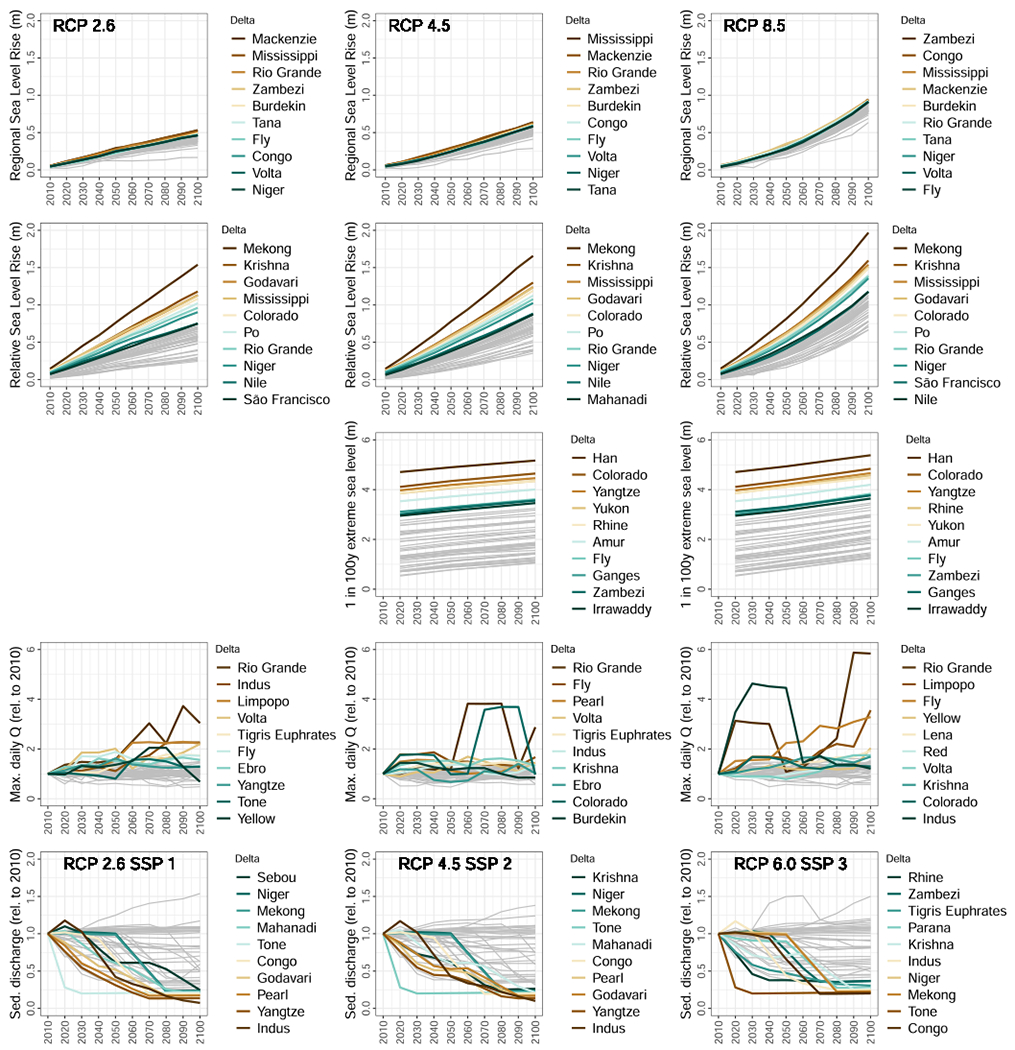
Geophysical pressures on individual deltas under three future climate scenarios. By row (top to bottom): sea-level rise (median of model ensemble) off the coast of the deltas analysed; relative sea-level rise including land subsidence; extreme sea levels (1 in 100 y events); 30-year maximum daily discharge (model ensemble average; change relative to 2010); 30-year average annual sediment discharge (change relative to 2010). Note that extreme sea level estimations are given for current, 2050, and 2100, and we interpolate here. Top-10 rivers are shown for each panel (bottom-10 for sediment). All other deltas are shown as grey lines. No extreme sea level estimations are available for RCP2.6. See Materials and Methods for full details of data sources and processing.

Sea levels are projected to continually rise with similar trends for all deltas analysed, with the exception of the Vistula delta (Baltic Sea) and Tigris Euphrates (Persian Gulf), which have noticeably lower curves. The highest SLR is seen in African and North American deltas under all three scenarios (Fig. 5). Deltas draining to inland seas (Danube and Dnieper—Black Sea; Volga—Caspian Sea) are not included. Under RCP2.6 the highest rise in sea level by 2100 is 0.53 m for the Mackenzie delta (Canada); under RCP4.5 it is 0.64 m for the Mississippi delta; and under RCP8.5 the highest SLR is over 0.94 m for the Zambezi (Mozambique) and Congo (Angola/Democratic Republic of the Congo) deltas.

Compared to regional SLR off the coast, land subsidence is a far greater contributor to relative SLR in most deltas (Minderhoud et al., 2020; Nicholls et al., 2021). By extrapolating the annual average delta subsidence rates of Nicholls et al. (2021) (updated from Ericson et al. 2006) and adding this to regional SLR off the coast of each delta, we see more than 0.9 m of relative SLR by 2100 for seven deltas under RCP2.6, and more than 1.3 m in these seven deltas under RCP8.5 (Fig. 5). As for regional SLR, these amounts of relative SLR by the end of the century dwarf those seen by 2030. Four of the top five deltas in terms of relative SLR (Mekong, Krishna, Godavari, and Colorado) are not among the worst cases when we look at regional SLR alone (Fig. 5), indicating the absolute necessity to place land subsidence management and mitigation (e.g., regulation of groundwater extraction and sedimentation with tidal river management; Islam et al., 2020; Minderhoud et al., 2020) at the centre of sustainable development in some of the most important deltas globally. Because of the paucity in subsidence data worldwide, for a number of deltas we adopted an estimated rate of 1 mm/y (following Nicholls et al., 2021). Simply judging by the higher rates of subsidence attributed to delta with data available, this assumption may well be underestimation of the real situation for a number of these deltas, creating an unknown but potentially large uncertainty in our assessment and ranking of deltas. Moreover, as subsidence can accelerate rapidly in a considerably short time, as shown by the case of the Mekong delta where groundwater extraction-induced subsidence rates increased from several mm/y in the 90th to several cm/y in the last decade (Minderhoud et al., 2017). Additionally, as we used delta-wide average subsidence rates, we underestimate the effect of relative SLR in local subsidence hotspots, such as certain cities within or at the edge of deltas, which can be a magnitude higher than overall delta subsidence rates in our set of 49 deltas (e.g., up to 28.5 mm/y in Bangkok region compared to 1.5 mm/y in the Chao Phraya delta where the city is located; Nicholls et al., 2021).

On top of regional and relative SLR, extreme sea levels pose particularly large threats to deltas because their elevations are only a matter of metres above mean sea level. Extreme sea levels (1 in 100 y) currently range from around 0.5 m to 4.7 m for the set of deltas. The Han delta (North and South Korea) has by far the most extreme sea levels currently and in the future, reaching almost 5.4 m by 2100 under RCP8.5 and only marginally less under RCP4.5. In addition to the Han, five deltas also have substantially higher extreme sea levels than those remaining (Fig. 5). The magnitude of extreme sea levels is much higher than SLR, but these are rare events, and they are superimposed on top of SLR, which inevitably makes them worse.

River discharge is the other key driver of high water levels in deltas. The projections of (model ensemble average) 30-year maximum daily water discharge show large variations over time (Figs. 4 and S4), but with 25 of the deltas showing an increase in all scenarios by the end of the century compared to only five showing a decrease. The magnitudes also skew towards higher relative increases than decreases in every scenario (Fig. S4), with the largest being an almost six-fold increase in the maximum for the Rio Grande delta (USA and Mexico) by 2090 under RCP8.5 (Fig. 5). In addition to the Rio Grande, the Indus (India and Pakistan), Limpopo (Mozambique), and Volta (Ghana) have more than a two-fold increase in maximum discharge by 2100 under RCP2.6; while the Limpopo, Fly (Papua New Guinea), and Yellow (China) have so under RCP8.5 (Fig. 5). The Rio Grande is consistently highly variable from decade to decade across all scenarios, while the Colorado delta (Mexico) is also highly variable under RCPs 4.5 and 6.0, as is the Indus under RCP8.5, particularly in the first half of the century (Fig. 5).

While floods can be a destructive hazard they are also essential for functioning delta systems, delivering water, sediments, and nutrients that support ecosystems and agriculture in the delta plains. Thus, a reduction in flood extents will have consequences for delta functioning. Reductions in maximum daily flows of more than 50 % are seen in the model results for the Sebou (Morocco) for three of the four scenarios (excluding RCP6.0) and São Francisco (Brazil) for two scenarios (RCPs 2.6 and 6.0). Four deltas have a reduction of more than 20 % across all scenarios, including the Sebou, São Francisco, Chao Phraya (Thailand), and Moulouya (Morocco) (Fig. S4). A total of 12 deltas have a reduction of more than 20 % in RCP4.5, followed by 11 in RCP2.6, nine in RCP6.0, and seven in RCP8.5. The largest reduction in maximum daily discharge is a 64 % decline by 2100, relative to 2010, for the São Francisco under RCP6.0. The earliest decline by more than 50 % relative to 2010 is seen by 2040 for the Sebou under RCP8.5.

Another fluvial aspect crucial for deltas is sediment load (Fig. 5). The majority of the deltas (28) are projected to see a decrease in sediment over the 21st century, regardless of the scenario, with 22 deltas having a decrease of over 50 % in at least one scenario, and only 12 of the deltas projected to experience increases in all scenarios. The decreases are caused by reservoir construction and increases in wealth, which correlate with other water engineering and influence land use, reduce soil erosion and river sediment loads. The largest increases over the century are seen in the Limpopo delta (Mozambique), which has no projected dam construction or significant socio-economic change that would reduce sediment loads, and the river is particularly sensitive to the remaining driver, climate change, which is the force behind all other increases in sediment loads over the century. Some rivers show more complex trends caused by interactions between the timing of different drivers. For instance, increases in agricultural production driven by population and economic growth may cause an increase in erosion and sediment delivery. At the same time, rising wealth levels may enable agricultural practices that reduce soil erosion for the benefit of agricultural production, possibly reducing sediment delivery from drainage basins. These complexities also lead to the rank order of deltas according to their sediment change being highly variable across the three scenarios; in particular, the four deltas experiencing the greatest sediment loss for RCP8.5-SSP3 are different from the other two scenarios (Fig. 5).

The different geophysical determinants of risk and (un)sustainability in deltas differentiate the deltas to varying degrees. The difference in regional SLR among deltas is much smaller than that for relative SLR, particularly by 2100 (Fig. 5), indicating that land subsidence rates play a much greater role in differentiating risk among deltas than does global climate change via regional sea-level rise. Extreme sea levels even further differentiate delta risk (with values spanning more than 4 m difference, compared to a maximum difference between deltas of around 1.3 m by 2100 for relative SLR). However, extreme sea levels are beyond the control of delta countries, whereas human-induced land subsidence, responsible for the highest rates, is controllable (e.g., successful mitigation of land subsidence in cities like Tokyo (Kaneko and Toyota, 2011) and Bangkok (Lorphensri et al., 2011)). On top of sea levels, these 49 deltas are substantially differentiated based on the degree of sediment deprivation relative to 2010 loads, with the Indus (India and Pakistan) losing 93 % by 2100 while the Limpopo (Mozambique) gains 54 %, under RCP2.6-SSP1 (Fig. 5). Although, many of these rivers are already currently starved of much of their historical sediment load (Dunn et al., 2019), so the extent to which further declines would determine the (un)sustainability of deltas in the future remains uncertain.

#### 3.2.3. Socio-economic conditions in delta countries

**Fig. 6.**
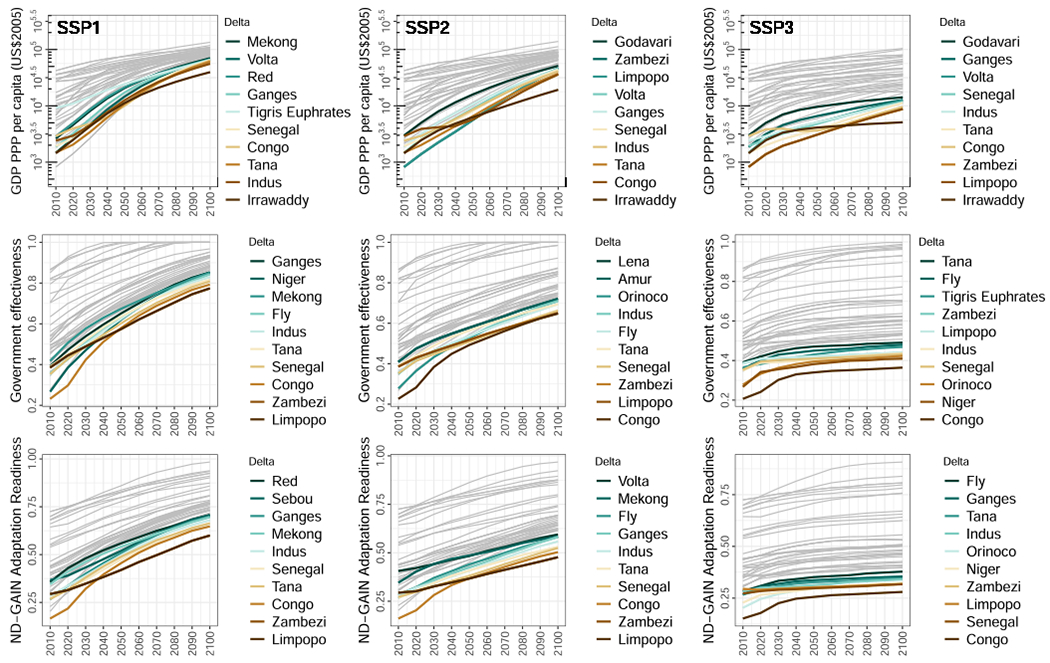
Future socio-economic developments affecting delta risk under three future development scenarios. By row (top to bottom): GDP (PPP) per capita; Government effectiveness from the World Bank’s Worldwide Governance Indicators; Notre Dame Global Adaptation Initiative (ND-GAIN) index of adaptation readiness. We use GDP and GDP per capita as crude indicators of wealth, which we interpret to reduce vulnerability through increasing the capacity to invest in adaptation strategies and the quality of infrastructure, following Tessler et al. (2015). Thus, we highlight the bottom ten (most vulnerable) deltas to emphasise where the main challenges lie. However, GDP located in the delta can also increase risk by increasing infrastructure and capital exposure to hazards. All other deltas are shown as grey lines. All indicators are calculated at the country level for those countries in which each delta is located. For deltas spanning multiple countries, we calculate the spatially-weighted average based on the fraction of delta area within each country. Please see methods for full details on calculations. Note Irrawaddy (Myanmar/Burma) missing data for government effectiveness and adaptation readiness.

Economic wealth, government effectiveness, and readiness and capacity to adapt to environmental change will determine, to a large extent but not completely, the ability to manage future risk in deltas (Tessler et al., 2015) and, thus, the success or failure of SDGs in these places. We use GDP as a crude indicator of wealth, but we also note that GDP located in deltas can actually increase risk because of the increase in capital exposed. Our analysis of the countries within which our set of deltas are located suggests that wealth, government effectiveness, and adaptation readiness could improve in the future under all scenarios, but that the amount of improvement greatly depends on the scenario and the present level from which each delta country develops (Fig. 6). The single exception of a declining indicator in our results is the Irrawaddy delta (Myanmar/Burma) under SSP4, whose GDP declines after 2030 to a level below that of 2010 (Fig. S5). This reflects the narrative of the SSP4 scenario—i.e., of a deeply unequal world (Calvin et al., 2017)—along with the present reality of prolonged political instability and hampered economic development in Myanmar/Burma (Jong-A-Pin, 2009). This combination will undoubtedly create extreme challenges for adaptation in the Irrawaddy delta, should this scenario eventuate.

The greatest improvement in GDP, government effectiveness, and adaptation readiness is seen under SSP1 and SSP5. Under SSP1, per capita GDP (in purchasing power parity) in delta countries rises to over US$39,000 (in 2005 USD equivalent) by 2100 even for the least developed delta country (Fig. 6). The same metric reaches almost US$68,000 for all delta countries under SSP5, of course with added challenges because of limited mitigation of greenhouse gas emissions in this future (O’Neill et al., 2017). There is strong convergence of per capita GDP and government effectiveness in delta countries under SSP1 and SSP5 (Figs. 5 and S5), contrasted by the widening gap between developed and developing delta countries in SSP4 (Fig. S5). Compared to SSP1 and SSP5, improvements in the indices are slower for SSP2 and SSP3, with only marginal increases in government effectiveness and adaptation readiness, in particular, under SSP3 (Fig. 6).

Those delta countries with consistently the lowest scores for all these indices are in Africa or South and Southeast Asia, with the addition of the Fly (Papua New Guinea) and the Orinoco (Venezuela) deltas. However, the relative position of several African deltas in particular (e. g. the Zambezi and Limpopo deltas) among the lowest values is highly variable depending upon scenario and indicator (Fig. 6). The Tigris Euphrates (Iran, Iraq, Kuwait) and Vietnamese deltas (Mekong and Red) also appear among the lowest scores in 2100 for several indicators and scenarios. Note that no data on government effectiveness or adaptation readiness was available for the Irrawaddy delta, which may in fact be the lowest overall for these indicators as it is for GDP.

## 4. Discussion and recommendations

### 4.1. Sustainable development and future risk in deltas

Our results confirm previous assertions by other scholars (e.g., Nicholls et al., 2019; Szabo et al., 2016) that deltas are important places in the context of global sustainable development. High population density, rapid urbanisation, and critical agricultural land mean many deltas are places of particular importance for creating healthy and prosperous communities, safe and sustainable cities, and ensuring food security. Our analysis of plausible global futures shows that these attributes of deltas will become more important over the century as they will require more area and pose more and stronger trade-offs, with their development being heightened beyond the 2030 horizon of the SDGs. At the same time, deltas are precariously positioned between upstream development, changes in water and sediment flows into the delta, and rising seas (Dunn et al., 2019; Nicholls et al., 2021; Schmitt et al., 2018), which will continue to exert pressure on the safe operating spaces of these important systems. These trends, too, are expected to increase well beyond 2030, unless (but perhaps even if) urgent management action is taken—for example, global mitigation of greenhouse gas emissions to slow SLR; local mitigation of groundwater extraction to slow land subsidence (e.g., Minderhoud et al., 2020); and sedimentation strategies to maintain and raise delta surface elevation (e.g., Dunn and Minderhoud, 2022).

Maintaining the ecosystem service of food provisioning from fertile delta lands is of particular importance when considering delta risk and the SDGs. Many of the world’s deltas are already saturated with cropland, which limits the physical space for other land use types (e.g., the land required for urbanisation or for mitigation or adaptation activities; Nicholls et al., 2019) and creates tensions and trade-offs with the ecological functioning of deltas. Model output suggests that under all SSP baseline scenarios there is a considerable demand for cropland worldwide, with the productive land in deltas being in high demand. (It should be noted that because we use SSP data from different sources, there can be slight inconsistencies among these sources, but high numbers for urban and agricultural land from different sources are indicative of multiple claims on land.) Those deltas almost entirely used for irrigated agriculture (e.g., the Red and Yangtze) face additional challenges for implementing and successfully achieving the SDGs. For example, the tension between food production and water consumption is a trade-off that must be managed appropriately in any river basin, but the geomorphic and hydrological consequences of irrigation water withdrawals (particularly groundwater; Minderhoud et al., 2020) that play out in deltas create an additional layer of complexity. Alternative scenarios beyond those analysed here show that the need for cropland can be reduced by increasing yields, reducing food waste, or decreasing conversion to animal calories (Leclère et al., 2020; Stehfest et al., 2019). Transforming to sustainable food systems globally, and in deltas specifically, thus requires consideration of coupled environmental and social systems as wholes (Sachs et al., 2019), rather than pursuit of increasing agricultural productivity within a growth paradigm.

A range of pressures from within and outside of deltas and from various causes affect the long-term sustainability of these systems, and our results show change in all elements of delta risk (sensu Tessler et al., 2015). For many deltas, land subsidence is a far greater driver of relative sea-level rise than rising seas themselves (Nicholls et al., 2021), and several of those same deltas could be starved of almost their entire current fluvial sediment load within decades (Dunn et al., 2019). Land is already maximally used in many deltas, creating a “locked-in” state (Santos and Dekker, 2020) lacking resilience to deal with change and shocks to the system. New risks could also emerge in certain deltas through urbanisation and increasing irrigated agriculture in regions where these land uses did not previously dominate. On the other hand, strengthening institutions and increasing wealth are apparent in all scenarios, which, although uncertain, give hope that the adaptive capacity of societies living in deltas will also increase, particularly under SSP1 (Andrijevic et al., 2020). However, institutions may remain weak in many countries, particularly under SSP3 and SSP4 (Andrijevic et al., 2020), and limited space for adaptation in many deltas will likely hinder progress. Combined, these developments (or lack thereof) could create soft and hard limits to adaptation, respectively. These findings reveal limitations to the temporally-stationary delta risk assessment of Tessler et al. (2015), indicating that 1) the relative change in delta risk will depend on the future development pathway; 2) the deltas most at risk today may not be so in the future; and 3) the determinants of risk for which delta countries have agency to mitigate and adapt will vary by delta and future development pathway.

The concepts of resilience, adaptive capacity, transformation, and “lock-in” (sensu Santos and Dekker, 2020) are highly relevant for a discussion of future risks to deltas and their sustainable development pathways. Deltas have inherent (ecological) adaptive capacity, which is the potential for deltas to be resilient (see Angeler et al., 2019), and (social) adaptive capacity in laws, institutions (Garmestani et al., 2019) and organizations (Vallury et al., 2022). The dynamics of cross-scale and cross-level interactions in deltas are affected by institutions and organisations operating at multiple levels and scales, which has important ramifications for governance of deltas (Cash et al., 2006). Delta governance has primarily been influenced by engineering, community, and disaster resilience, focusing on return and recovery responses to environmental change and procuring a reliable flow of a small set of ecosystem services for humans (e.g., flood protection, water supply). Over time this form of delta governance has reduced the resilience of many deltas (lock-in or traps), limiting options for adaptation or in more forward-looking cases, transformation of delta social-ecological systems (Jozaei et al., 2022). Incorporating social-ecological resilience and its core aspects (i.e., panarchy, adaptation, and transformation) into delta governance can help to move deltas out of lock-in by identifying capacities and pathways for adaptation or transformation, as well as accounting for scale and cross-scale interactions. How institutional and legal dimensions of governance develop in the future is, thus, highly important for the resilience and sustainability of deltas in the future (see, e.g., Du et al. (2022) on legal and governance dimensions of the climate adaptation “solution space”).

Our results show that the future risks faced in deltas depend highly upon the development pathway taken (e.g., SSP1 vs. SSP3 or RCP2.6 vs. RCP8.5), the component of risk (e.g., regional SLR vs. land subsidence), and the contingent geography of the delta (e.g., the highly developed and locked-in Rhine vs. the rapidly urbanising Niger delta). The large differences between scenarios and components of risk indicate a high degree of human agency over the future of deltas exists from local to global scales. The greatest challenges may lie in Asian and African deltas, where populations are high and climbing, delta lands are often saturated with agriculture, and financial wealth, governance capacity, and adaptation readiness lag behind other parts of the world. But despite the broad trends, deltas outside Asia and Africa are not immune from increasing risks, particularly regarding relative SLR (e.g., the Mississippi and Po deltas); although, in many cases the European deltas analysed are already sediment-starved and/or have in place infrastructure that allows managing for increasing water discharges.

Our results are, however, contingent upon the set of 49 deltas analysed, their extents delineated in each delta shapefile, and the models and datasets used to source the indicators. The 49 deltas analysed for this research include the most populated and largest coastal deltas in the world, as well as a range of smaller and less populated deltas to provide a representative sample of the world’s deltas across climates, biomes, and socio-economic development states. We do not include all small and medium sized deltas partly due to the difficulty of defining deltas and delta areas at these scales and acquiring other appropriate data, so parts of our analysis of “non-delta” areas will include some delta areas, although these make up a vanishingly small fraction of our defined nondelta areas. We must also note that the non-delta area (i.e., the rest of the ice-free land surface) contains large tracts of extremely sparsely populated land with little to no cropland (e.g., the Sahara Desert, Central Australia, the Amazon Basin, Siberia), which undoubtedly deflates the statistics shown in Fig. 3 for the “rest of land”. Similarly, a potential side effect of including this range of delta areas in our dataset is that where the 49 deltas are averaged, the larger deltas have a disproportionate effect on the results due to their size. In particular, the five largest deltas make up almost half of the total land area in our set of 49 deltas. Nonetheless, we consider these a useful and interesting sample of deltas, which aligns with previous research (Tessler et al., 2015; Dunn et al., 2019; Nicholls et al., 2021). Future research could include analysis of deltas based on their attributes, for instance comparing deltas with low population density to those with high population density, and investigating any potential differences in their risk factors and development pathways. More deltas could also be included in analyses, for instance considering the datasets of Reader et al. (2022) and Nienhuis et al. (2020). Additionally, our results show how development and environmental change consistent with global storylines and pathways (i.e., the SSPs and RCPs) could place pressures on deltas, but not the locations at which any particular land use change might occur in any particular delta nor where land might be lost. The latter, require delta-scale simulation modelling (e.g., Hutton et al., 2018), which could use our data as global change boundary conditions in local models that dynamically simulate the interactions between, for example, relative SLR, channel migration, shoreline change, land use, and adaptation strategies (e.g., defend, retreat, advance). Indeed, incorporating adaptation strategies into future IAM simulations is a pressing task for modelling teams, and deltas may provide excellent model systems to experiment with.

### 4.2. Implications for global sustainable development agendas

While deltas and other hotspots (e.g., small islands, semi-arid regions, mountain areas) are important places for global sustainable development, the SDG evaluation framework is at the country level, which does not align with the boundaries and processes of the environmental systems (e.g., deltas) underpinning development (Kulonen et al., 2019; Scown, 2020). That is, the global institutions designed to govern and guide sustainable development do not fit with local environmental systems within which development and trade-offs take place and where risks manifest. Also within current institutional frameworks in most places, social and economic goals are prioritised over environmental ones (Craig and Ruhl, 2019; Custer et al., 2018; Forestier and Kim, 2020), and scholars have argued that environmental damage will not be avoided by pursuing the SDGs (Zeng et al., 2020). As a result, key social-environmental feedbacks are missed and remain key knowledge gaps for the 2030 Agenda (Mastrángelo et al., 2019; Reyers and Selig, 2020). This is particularly relevant in deltas, where many development pressures and environmental processes coalesce to impose severe risks on future sustainable development in these important places.

Scholars and practitioners of the SDGs should be cognisant of temporal and spatial scales, environmental feedbacks (Mastrángelo et al., 2019; Reyers et al., 2017; Reyers and Selig, 2020), upstream–downstream (and other distant or telecoupled; Xu et al., 2020) processes, and institutional fit for the physical systems within which sustainable development must occur. Our results suggest that 2030 may be short-sighted for SDGs in deltas, depending upon which future comes to pass, and that environmental feedbacks operating over decades may undermine social and economic development goals in deltas. Land subsidence exacerbated by groundwater extraction for economic development and irrigated agri- and aquaculture is one example (Minderhoud et al., 2017). Hydropower dams for renewable energy, which withhold the sediment required for deltas to maintain their elevation under relative SLR, is another (Dunn et al., 2019; Schmitt et al., 2018). Implementation of and progress towards the SDGs should be carefully considered within the boundaries of the physical systems they affect (Kulonen et al., 2019; Reyers and Selig, 2020; Szabo et al., 2015), not only within the political boundaries through which they were negotiated.

Greater conceptualisation and prioritisation of “the environment” is required in implementing the SDGs to 2030 and in future iterations of global sustainability goals. Currently, the environment is underprioritised in national SDG implementation (Custer et al., 2018; Forestier and Kim, 2020) and we argue here that environmental processes ignored in the SDGs may actually undermine them, unless they receive greater attention particularly in contexts such as deltas. Global agendas need to be localised in practice (Moallemi et al., 2019), but so far localisation of the SDGs is focused on socio-economic not environmental context. Focusing on “hotspots” for the SDGs can be an efficient way to concentrate resources for the greatest impact, but hotspots must be determined not only on societal needs but also environmental conditions (Szabo et al., 2016), particularly in the face of climate change, land degradation, and biodiversity loss. Cities, for example, may be hotspots for sustainability goals in the so-called “urban century” (Elmqvist et al., 2019), but cities in hotspots of environmental change, such as deltas, have a compounded risk.

Finally, Integrated Assessment Models (IAMs) have an important role to play in hypothesising and testing the effects of policies and actions on the SDGs (van Soest et al., 2019). We have analysed global IAM scenarios to explore how global development could affect key pressures on deltas for the SDGs. However, delta-scale processes such as land subsidence, channel migration, and shoreline change are not considered in regional or global IAMs, and we have also treated them in a simplified manner here. Delta- and other context-specific IAMs are clearly required to successfully implement the SDGs and balance trade-offs in local SDG hotspots (e.g., Hutton et al., 2018). These could be externally “forced” consistent with the global SSP-RCP framework using the data resulting from our analyses. Ultimately, though, processes across scales that affect the sustainability of social-ecological systems in deltas and other hotspots should be dynamically simulated. Better incorporation of environmental processes and feedbacks into regional and global IAMs will help improve their utility for global sustainability goals (van Soest et al., 2019; Van Vuuren et al., 2012). For example, when people, cities, and cropland are allocated to land grid cells that are seriously at risk of being drowned by 2100 or earlier, this feedback should be taken into account and may have dramatic consequences for food production and migration in the models and in our real world. Similarly, adaptation strategies such as defend, retreat, or advance could be simulated to explore different adaptation scenarios in IAMs, which currently lag far behind mitigation scenarios in global models. Socio-economic shocks (e.g., COVID-19) and Earth system tipping points create further complexities affecting delta risk and global sustainable development, yet these, too, are not adequately accounted for in global IAM simulations, although addressing them remains a model development priority (Franzke et al., 2022; Hanna and Gross, 2021; Kopp et al., 2016). Such dynamic interactions between coupled environmental and social systems across scales should be high on the research agenda for IAM, delta, and other sustainability research communities so that we can explore and prepare for different risks and pathways via which to achieve the SDGs and sustainable development beyond 2030.

## Supplementary Material

Supplement1

## Figures and Tables

**Table 1:** Indicators calculated for each delta. Indicators are chosen either directly or indirectly related to the components of delta risk as defined by Tessler et al. (2015). Please see details of our calculations in text. See Table S1 from Tessler et al. (2015) for details on their indicators and rationale.

Indicators calculated for each delta	Source	Related indicators measured by Tessler et al. (2015)	Delta risk indices from Tessler et al.
Population density	Jones and O’Neill (2016)	Population density	Anthropogenic Conditioning Index (ACI)
Urban land fraction	Gao and O’Neill (2020)	Impervious surface area Wetland disconnectivity	
Cropland fraction (total and irrigated)^1^	IMAGE^2^	Wetland disconnectivity Groundwater depletion	
Relative change in sediment delivery from basin to deltas	Dunn et al. (2019)	Reservoir volume sediment trapping	
Rate of regional sea-level rise	Pörtner et al. (2019)	Sea-level rise trend	
Rate of land subsidence	Nicholls et al. (2021) Cenni et al. (2021)	Groundwater depletion Oil and gas extraction	
Relative change in 30-year maximum daily discharge	PCR-GLOBWB^3^	30-year river discharge	Hazardous Event Index (HEI)
Extreme sea levels^4^	Kirezci et al. (2020)	30-year wave energy M_2_ tidal amplitude Tropical cyclone frequency	
Aggregate GDP	Dellink et al. (2017)	Aggregate GDP	Investment Deficit Index (IDI)
Per capita GDP	KC and Lutz (2017)	Per capita GDP	
Government effectiveness	Andrijevic et al. (2020)	Government effectiveness	
Adaptation readiness^5^	Andrijevic et al. (2020)	*n/a*	*n/a*

Notes:

1 Cropland area was not at all considered by Tessler et al. (2015)—we interpret it to relate indirectly to their variables of wetland disconnectivity and groundwater depletion (particularly irrigated cropland), but it is also highly relevant for an analysis of delta sustainable development in its own right;

2 Integrated Assessment Model IMAGE 3.0;

3 Global hydrological model PCR-GLOBWB;

4 We assume a measure of extreme sea levels captures the combined effects of wave energy, tidal amplitude, and storm surges, yet we acknowledge it does not account for cyclone wind gusts;

5 We include the indicator of adaptation readiness, which was not included by Tessler et al. (2015), because it is an indicator specifically constructed for the context of climate risk, as opposed to GDP and government effectiveness, which are generic.

## Data Availability

Data are provided in full in the supporting information.
