# Supplementary material for: Global change scenarios in coastal river deltas and their sustainable development implications
Source: Glob Environ Change. Author manuscript; Available in PMC 2024 Sep 1. (PMC10483986; doi:10.1016/j.gloenvcha.2023.102736)
Supplement: Supplement1 [file NIHMS1925337-supplement-Supplement1.zip › 1-s2.0-S0959378023001024-mmc1.docx]

Supplementary material

**Global change scenarios in coastal river deltas and their sustainable development implications**

**Supplementary Results**

Fig. S1 shows the delta-average population density and land use data from Fig. 2 in the manuscript (panels A-D) but with additional panels E-H showing the trends relative to 2010.

Figs. S2 and S3 show the individual delta population density and land use data from Fig. 3 in the manuscript but for all scenarios and with different deltas highlighted, depending upon amount of change or absolute value of cropland fraction.

Our results show slight inconsistencies among the different model outputs in terms of land use; for example, by 2100 the Yangtze delta has over 35% urban area and over 80% cropland under SSP2, which would be impossible. These inconsistencies are due to different modelled data used, and differences in grid cell size among the outputs (5 arcminutes compared to 1/8^th^ degree), but overall, these are negligible. While using different models for urban land and cropland means the different land uses are not necessarily harmonised and do not always sum to 100%, we chose to use the urban areas from Gao and O’Neill (2020) instead of those from IMAGE because of particularly low estimates of urban land in some Asian deltas in the latter, when compared to satellite-derived current land cover estimates.

Fig. S4 shows the absolute value of the (model ensemble mean) 30-year maximum daily discharge for all four RCPs, along with the relative change from 2010 (shown in manuscript Fig. 4) for all scenarios and highlighting the top-10 and bottom-10 deltas.

Fig. S5 shows absolute GDP in addition to per capita GDP, government effectiveness, and adaptation readiness (shown in manuscript Fig. 5) for all scenarios.


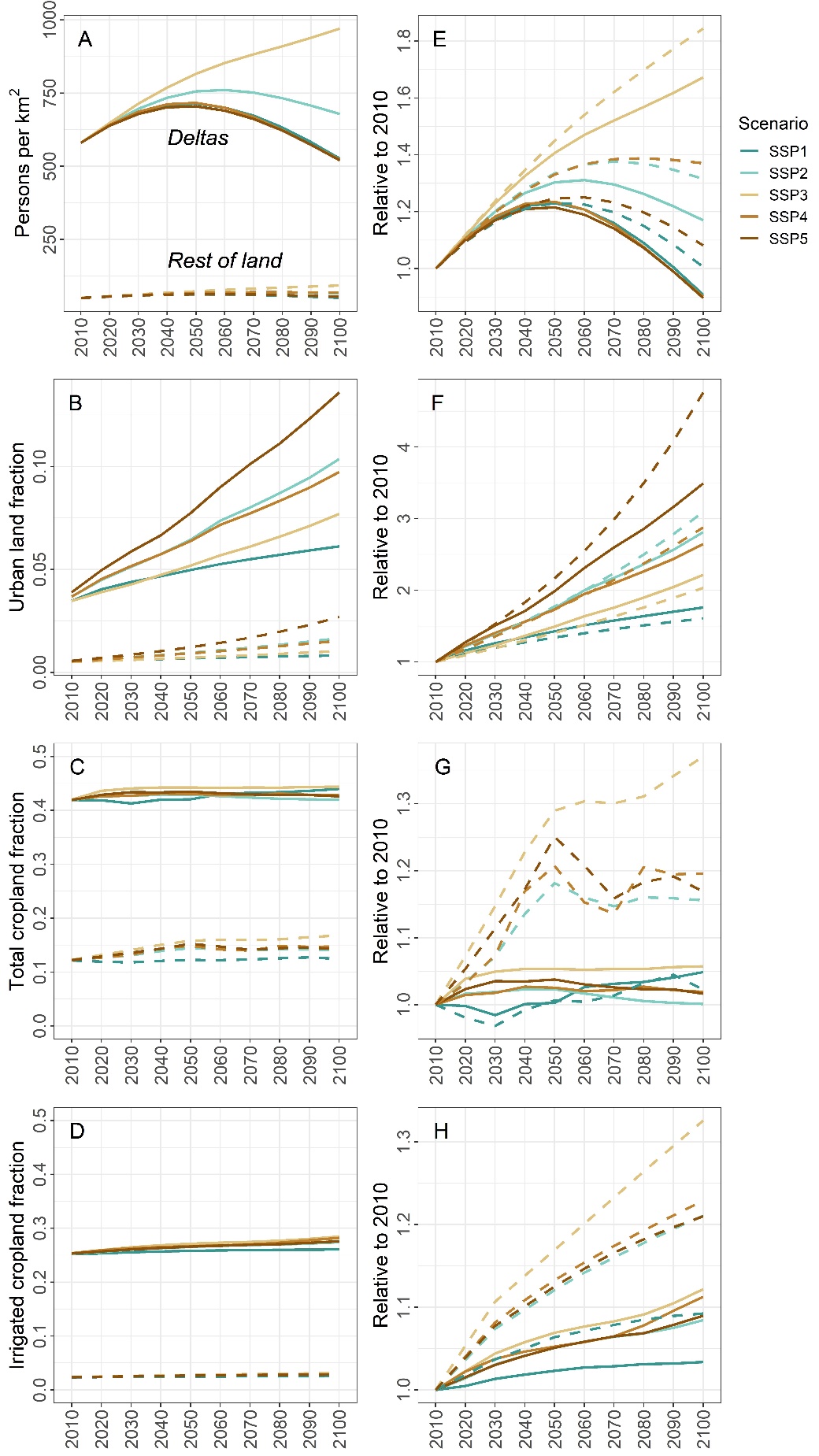


Fig. S1. Delta-average population density, urban land, and total and irrigated cropland fractions for all SSPs, shown in density/fractional terms over time in panels A-D and as change relative to 2010 in panels E-H.


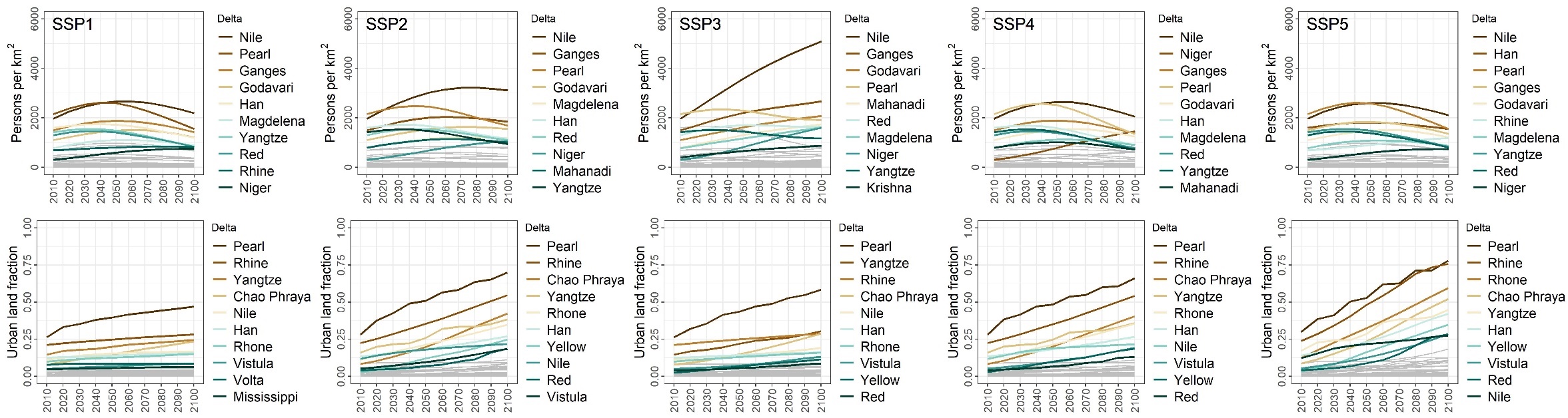


Fig. S2. Population density (top row) and urban land fraction (bottom row) in deltas under the five SSPs. Top-10 deltas in 2100 are highlighted.


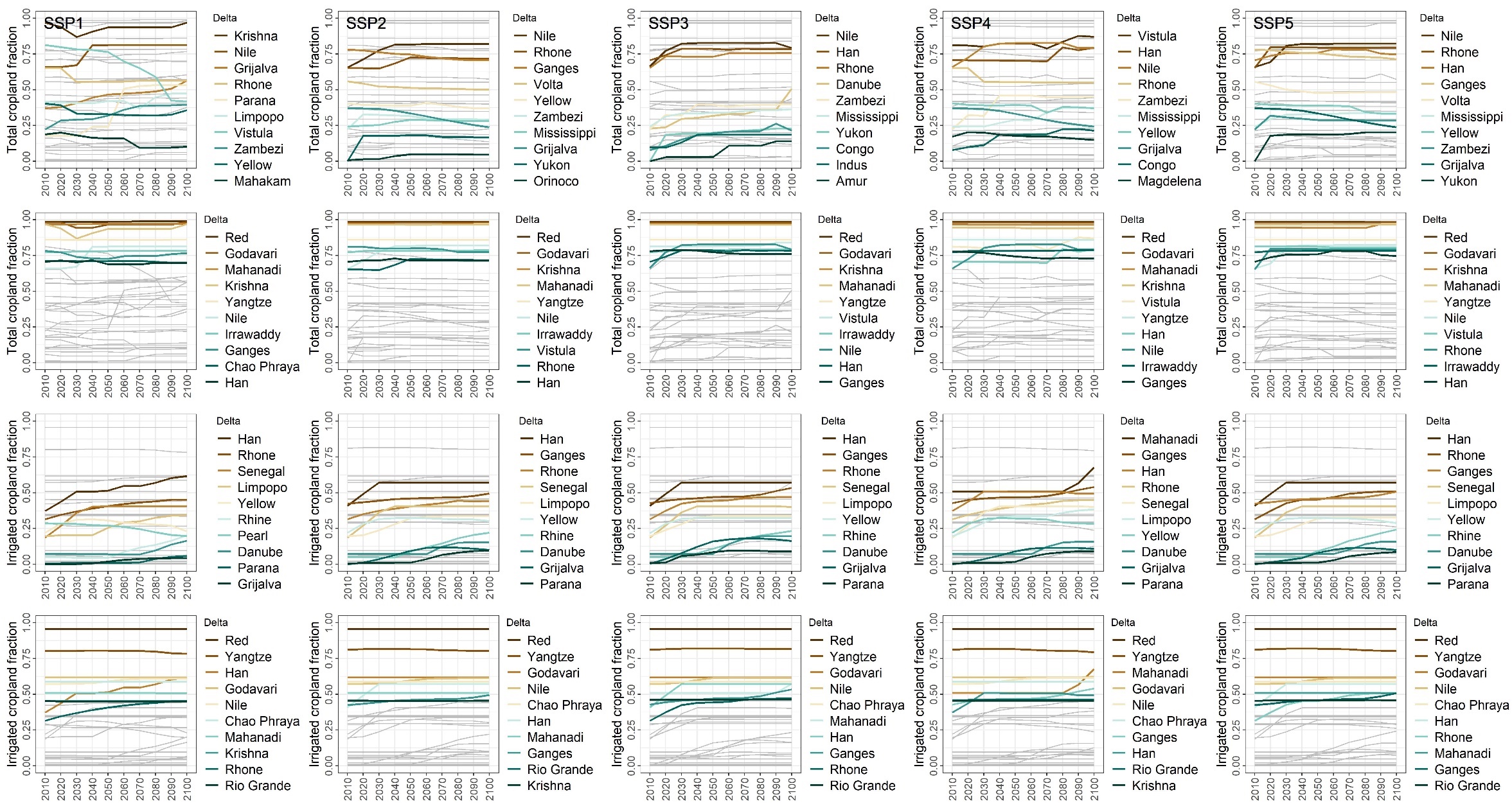


Fig. S3. Cropland fractions for all five SSPs. From top to bottom (by rows): total cropland fraction highlighting deltas with greatest change; total cropland fraction highlighting deltas with highest fraction in 2100; irrigated cropland fraction highlighting deltas with greatest change; irrigated cropland fraction highlighting deltas with highest fraction in 2100.


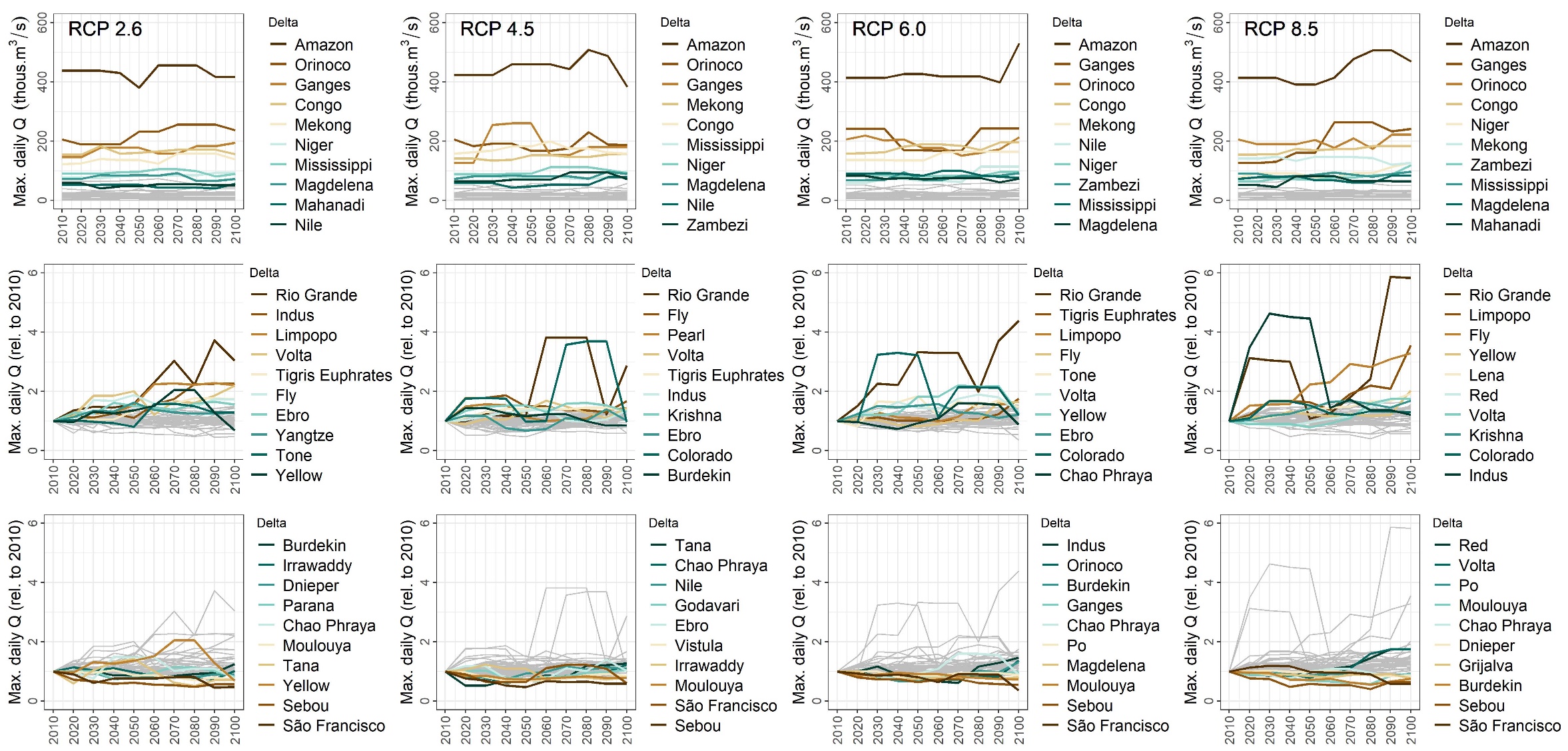


Fig. S4. 30-year maximum daily discharge for each river, average across the five climate models, for the four RCPs. From top to bottom (by row): Maximum daily discharge highlighting the ten highest in 2100; change in 30-year maximum daily discharge relative to 2010 highlighting the ten highest in any decade, coloured by rank order in 2100; change in 30-year maximum daily discharge relative to 2010 highlighting the ten lowest in any decade, coloured by rank order in 2100.


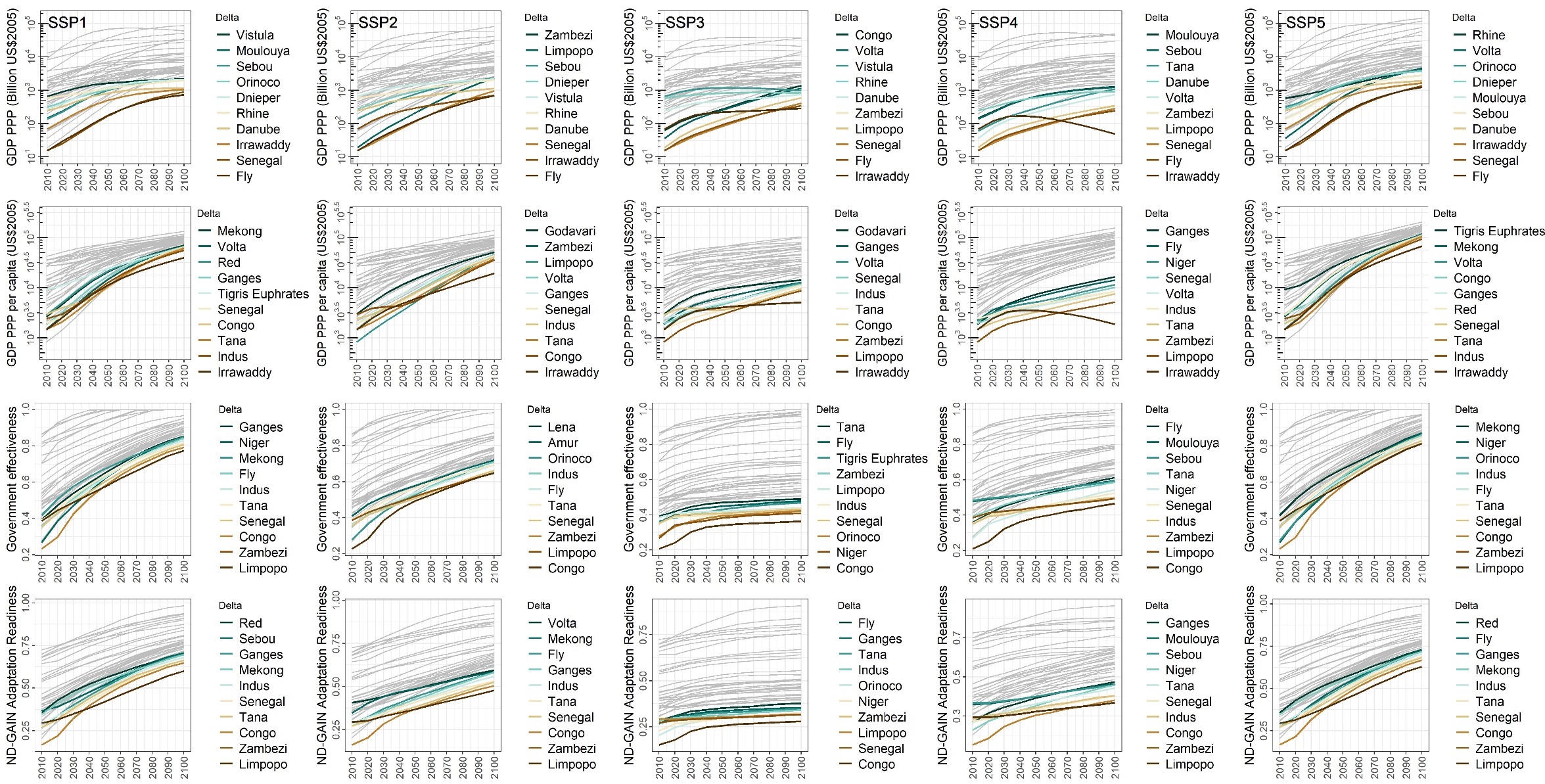


Fig. S5. Delta country economic, governance, and adaptation indicators for the five SSPs. From top to bottom (by row): GDP in purchasing power parity (PPP); GDP (PPP) per capita; government effectiveness from the World Bank’s Worldwide Governance Indicators; and the Notre Dame Global Adaptation Initiative (ND-GAIN) index of adaptation readiness.
